# The COVID‐19 Yorkshire Rehabilitation Scale (C19‐YRS): Application and psychometric analysis in a post‐COVID‐19 syndrome cohort

**DOI:** 10.1002/jmv.27415

**Published:** 2021-11-05

**Authors:** Rory J. O'Connor, Nick Preston, Amy Parkin, Sophie Makower, Denise Ross, Jeremy Gee, Stephen J. Halpin, Mike Horton, Manoj Sivan

**Affiliations:** ^1^ Academic Department of Rehabilitation Medicine, Leeds Institute of Rheumatic and Musculoskeletal Medicine, School of Medicine University of Leeds Leeds UK; ^2^ National Demonstration Centre for Rehabilitation Leeds Teaching Hospitals NHS Trust Leeds UK; ^3^ Leeds Community Healthcare NHS Trust Leeds UK; ^4^ Airedale Foundation NHS Trust UK

**Keywords:** long COVID, Patient Reported Outcome Measure (PROM), post‐COVID‐19 symptoms, psychometrics, SARS CoV‐2

## Abstract

As our understanding of the nature and prevalence of post‐coronavirus disease 2019 (COVID‐19) syndrome (PCS) is increasing, a measure of the impact of COVID‐19 could provide valuable insights into patients' perceptions in clinical trials and epidemiological studies as well as routine clinical practice. To evaluate the clinical usefulness and psychometric properties of the COVID‐19 Yorkshire Rehabilitation Scale (C19‐YRS) in patients with PCS, a prospective, observational study of 187 consecutive patients attending a post‐COVID‐19 rehabilitation clinic was conducted. The C19‐YRS was used to record patients' symptoms, functioning, and disability. A global health question was used to measure the overall impact of PCS on health. Classical psychometric methods (data quality, scaling assumptions, targeting, reliability, and validity) were used to assess the C19‐YRS. For the total group, missing data were low, scaling and targeting assumptions were satisfied, and internal consistency was high (Cronbach's *α* = 0.891). Relationships between the overall perception of health and patients' reports of symptoms, functioning, and disability demonstrated good concordance. This is the first study to examine the psychometric properties of an outcome measure in patients with PCS. In this sample of patients, the C19‐YRS was clinically useful and satisfied standard psychometric criteria, providing preliminary evidence of its suitability as a measure of PCS.

## 1 INTRODUCTION

The medium and long‐term problems experienced by survivors of coronavirus disease 2019 (COVID‐19) are emerging, with over one million people in the UK who contracted COVID‐19 reporting symptoms and functional problems more than 4 weeks after onset of the acute illness.^1^ Almost 700 000 people report ongoing impact on their health and functioning more than 12 weeks after the acute infection, and this combination of symptoms and functional difficulties is recognized as a new syndrome called “Long COVID” (LC) or post‐COVID‐19 syndrome (PCS).^1^ The most common symptoms include fatigue, breathlessness, pain, anxiety, and cognitive impairment, but there are over 200 reported symptoms affecting 10 organ systems.^2^ One study following 143 individuals seven weeks postdischarge found 53% of patients reported fatigue, 43% breathlessness, and 27% joint pain.^2^ A substantial number of people report limitations with their activities of daily living, with almost 130 000 patients stating that these limitations are severe.^3^ Given the novelty and uniqueness of the syndrome, it is unsurprising that standardized assessments of functioning, disability, and health are lacking. While generic assessments are available, these have been shown to lack responsiveness or be useful only for discriminative purposes.^4^, ^5^

The long‐term symptoms of COVID‐19 might be predicted from the previous coronavirus outbreaks in 2002 and 2012—Severe Acute Respiratory Syndrome (SARS) and Middle East Respiratory Syndrome (MERS), respectively. Our meta‐analysis of follow‐up studies demonstrated that 25% of hospitalized survivors of SARS and MERS experienced reduced lung function and lower exercise capacity 6 months postdischarge.^6^ One year on, posttraumatic stress disorder (PTSD), depression, anxiety, and reduced quality of life were observed. Preliminary research suggests that the impact of COVID‐19 infection is similar.^7^ Five studies in our metanalysis included the Medical Outcomes Trust Short‐Form 36‐item generic health outcome measure (SF‐36) as part of a basket of measures to try to determine health outcomes after the acute infection. The breadth of long‐term symptoms in patients affected by the previous SARS outbreaks, and the emerging evidence of the long‐term impact of COVID‐19, means that a single, generic health outcome measure, or indeed a basket of measures, will be adequate to capture the breadth of these symptoms in a succinct way that is acceptable to patients and clinicians. Furthermore, the responsiveness of the SF‐36 in detecting clinical change when it occurred was small, limiting the utility of this measure in measuring the effect of an intervention.

Using symptoms and functional difficulties of PCS that were being reported by survivors of acute COVID‐19 infection and the healthcare professionals involved in their care from across the clinical sites in the Yorkshire region,^7^ we developed a condition‐specific measure for PCS. The COVID‐19 Yorkshire Rehabilitation Scale (C19‐YRS) is a 22‐item patient‐reported outcome measure designed to evaluate the long‐term impact of COVID‐19 across the domains of Activities and Participation of the International Classification of Functioning, Disability, and Health and evaluate the impact of PCS rehabilitation.^8^ The C19‐YRS now includes clinician‐completed, self‐report, and digital versions.^3^ Content validity of the C19‐YRS has been demonstrated,^7^ and the C19‐YRS is now used in the UK's first specialist PCS community rehabilitation service and 26 other National Health Service (NHS) PCS services in the UK.^9^

This article describes the first stage in establishing the initial psychometric properties of the C19‐YRS as an outcome measure for PCS using classical test theory.^10^ Its ongoing development will investigate and address any problems with its psychometric properties using Item Response Theory (specifically, Rasch analysis).^11^ This will explore the presence of differential item functioning, local dependency of items, and will examine unidimensionality and the YRS' responsiveness.

## 2 METHODS AND DESIGN

This study was a prospective, observational study, and psychometric analysis of data captured from long COVID patients using the C19‐YRS questionnaire. Long COVID patients were recruited from a community‐based Long COVID clinic within one of the UK's largest metropolitan areas. Data were collected in the service as part of routine clinical evaluation and ethical approval was obtained for the secondary analysis of anonymized data collected for the primary clinical purpose, which had been completed. A favorable ethical opinion was received from the University of Leeds School of Medicine Research Ethics Committee in January 2021 (reference MREC 20‐041—Secondary analysis of C19‐YRS (COVID‐19 Yorkshire Rehabilitation Scale) data collected for recording Long COVID symptoms and functional disability).

### 2.1 Participants and recruitment

Data were collected from patients attending a community‐based PCS rehabilitation service covering the Leeds City Region, a mixed urban and rural district in the North of England with a population of approximately 850 000 people, which includes areas of significant social deprivation. Patients were referred by their General Practitioner (GP), Community Matron, or Respiratory Physiotherapy team to a PCS Community Rehabilitation Service and completed a self‐report C19‐YRS as part of initial triage. Initial eligibility was decided using the inclusion criteria. To participate, each participant met the criteria stated:

#### 2.1.1 Inclusion criteria

• Patients are referred into the Long COVID Community Rehabilitation Service by their GP, Community Matron, or Respiratory Physiotherapy team to a PCS Community Rehabilitation Service (this includes young people aged 16 and above). A positive antigen or antibody test was not required within the eligibility criteria as routine testing was not available at the time of commencement of data collection.
• Ability to complete a self‐report C19‐YRS as part of initial triage. Although literacy and language ability were not initially screened unless highlighted by the referrer, support to complete the C19‐YRS form was provided where necessary by a family member or carer, clinician, researcher, or a proprietary translation service used by the clinical service.
• Willing and able to consent for data to be used anonymously for research and/or service evaluation purposes. Consent was gathered via the first page of the C19‐YRS form and did not affect a patient's access to treatment. Data are already collected in the service as part of routine initial evaluation to form a functional baseline and are documented in the patient's electronic clinical notes.

#### 2.1.2 Exclusion criteria

- Inability to consent

### 2.2 The C19‐YRS

The C19‐YRS consists of 22 items with each item rated on an 11‐point numerical rating scale from 0 (none of this symptom) to 10 (extremely severe level or impact). The C19‐YRS is divided into four subscales (range of total score for each subscale): symptom severity score (0–100), functional disability score (0–50), additional symptoms (0–60), and overall health (0–10). The C19‐YRS can be freely obtained under license from the University of Leeds (https://licensing.leeds.ac.uk/product/c19-yrs-covid-19-yorkshire-rehabilitation-scale).

**Figure 1:**
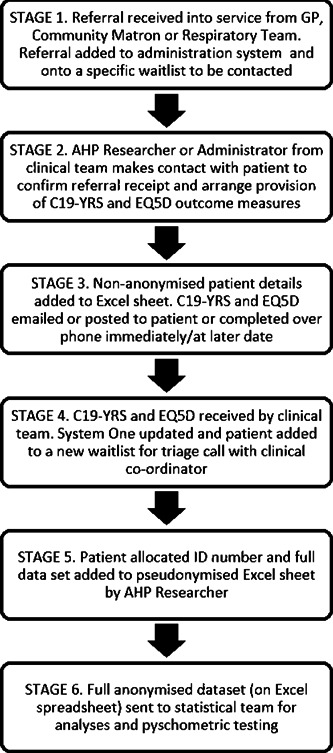
Flow chart illustrating patient recruitment and preparation of data for research purposes. C19‐YRS, COVID‐19 Yorkshire Rehabilitation Scale; GP, General Practitioner

At triage, the C19‐YRS was completed independently by each patient or, if the patient preferred, by a clinical researcher (an allied health professional seconded into a research position, who was involved in the initial assessment pathway) or a member of the clinical team via telephone. Patients' family members or carers were permitted to help complete the responses. On return of the C19‐YRS, the clinical researchers transferred the data from each completed C19‐YRS to an Excel spreadsheet. The data were fully anonymized, but details such as sex and age (not the date of birth) were included. The full process is illustrated in Figure 1. Anonymity was ensured by linking patient identifiable details to ID numbers on one Excel sheet, and full C19‐YRS data sets on another. Only the ID numbers linked the two documents, which were removed before statistical team input.

It was not felt by the research team that formal training was required before contacting patients (to organize the provision of the C19‐YRS) nor completing the measure via the phone, as it is possible to read questions verbatim from the C19‐YRS. A standard operating procedure and telephone script were agreed upon, including allowing questions to be repeated for clarification with prompts provided for scale grading.

### 2.3 Data analysis

Descriptive statistics are presented as an arithmetic mean and *SD*, or median and interquartile range as appropriate. Absolute and relative frequencies as appropriate for demographic and categorical variables on the C19‐YRS are presented. Analyses were carried out using IBM SPSS (Statistics 26, Release 26.0.0.0, 64‐bit edition, IBM Corp.). Four psychometric analyses (data quality, scaling assumptions, targeting, and reliability) were undertaken.

### 2.4 Data quality

Data quality concerns the extent to which a scale can be administered successfully in the target sample. The C19‐YRS data were examined for percentage missing items and the percentage of the sample for whom total scores could be calculated. For responders with missing items, imputed scores were not used.^12^

### 2.5 Scaling assumptions

Tests of scaling assumptions examine whether it is legitimate to sum item scores to generate scale scores. In order for a set of items to be legitimately summed to form a total score, a series of criteria should be satisfied.^13^, ^14^, ^15^ We tested the C19‐YRS against these criteria, which are:

Items should be roughly parallel, that is, measure at the same point on the scale and have similar variance, otherwise they do not contribute equally to the variance of the total score.^16^ A set of items is considered parallel when their item response option frequency distributions and their item mean scores and standard deviations are roughly similar.^14^
Items should measure the same underlying construct, otherwise, it is not appropriate to combine them to generate a total score.^11^ A set of items is considered to be measuring the same construct when each item's corrected item‐total correlation, which is the correlation between each item and the total score computed from the remaining items in that scale, exceeds 0.30.^16^
Items in the scale should contain a similar proportion of information concerning the construct being measured. This criterion is considered satisfied if the corrected item‐total correlations exceed 0.30.^17^

### 2.6 Targeting

Targeting refers to the match between the distribution of health problems in the sample and the range of health problems measured by the scale. The better this match, the greater the potential for precise measurement. Targeting was evaluated by examining floor and ceiling effects, score distributions, and skewness statistics. Floor effects are the percentage of patients scoring 10 (most severe impact of symptom) and ceiling effects are the percentage of patients scoring zero (symptom not present). It is recommended that floor and ceiling effects should be less than 20% each on each item.^18^

### 2.7 Reliability

Reliability describes the extent to which scale scores are free from random error. Scales should generate reliable estimates of the construct being measured (internal consistency). Cronbach's *α* coefficient was used to determine this criterion.^19^ Although a range of minimum values has been suggested, it is widely accepted that Cronbach's *α* should exceed 0.80 for group comparison studies.^15^

## 3 RESULTS

### 3.1 Sample characteristics

Data for the analyses were obtained from 188 consecutive assessments of PCS patients. Patient details are given in Table 1. One patient was removed from the analyses because a significant number of answers were missing, presumed to be an oversight of the respondent, or due to symptoms impacting on the ability to complete the scale in full. Follow‐up contact was not made with patients unless an answer to the “overall health” question was suspected to be erroneously scored (i.e., scoring their health higher post‐COVID than pre‐COVID).

**Table 1:** Patient demographics

	Non‐hospitalized	Hospitalized
Total no (%)	84% (*n* = 157)	15% (*n* = 28) ICU 5.4% (*n* = 10)
Age: mean (*SD*)	47.1 (*SD* 13.74)	51.9 (*SD* 12.83)
Duration of PCS in weeks: Average (*SD*); median (IQR)	24 (17); 30 (9–38)	18 (16); 12 (5–33)
Sex (%)		
Female	66% (*n* = 104)	43% (*n* = 12)
Male	34% (*n* = 54)	57% (*n* = 16)
Ethnicity (%)		
White‐British, Northern Irish, Rep of Ireland	80% (*n* = 126)	60.6% (*n* = 18)
Asian/British Asian	6% (*n* = 10)	18% (*n* = 5)
Black – Black British, African, Black African	2% (*n* = 3)	7.2% (*n* = 2)
Mixed – Asian, Mixed White and Black African, Mixed White and Black Caribbean	2.5% (*n* = 4)	0
Other – Undefined White, European or British	8% (*n* = 12)	7.1% (*n* = 2)
Arab	1.3% (*n* = 2)	1% (*n* = 3.6)
Occupation (%)		
Healthcare workers	30% (*n* = 47)	21% (*n* = 6)
Non‐healthcare	70% (*n* = 111)	79% (*n* = 22)
Impact on vocation (%)		
Reduced hours/adjusted work	17% (*n* = 26)	4% (*n* = 1)
Off‐sick	19% (*n* = 30)	57% (*n* = 16)
No effect	64% (*n* = 100)	39% (*n* = 11)
Symptoms in PCS (%)		
Fatigue	92% (*n* = 145)	89% (*n* = 25)
Noisy breathing	41% (*n* = 65)	54% (*n* = 15)
Cough/throat sensitivity	58% (*n* = 91)	68% (*n* = 19)
Pain (%)		
Chest pain	65% (*n* = 103)	61% (*n* = 17)
Muscle pain	70% (*n* = 111)	68% (*n* = 19)
Joint pain	59% (*n* = 93)	61% (*n* = 17)
Abdominal pain	31% (*n* = 49)	36% (*n* = 10)
Headache	70% (*n* = 110)	54% (*n* = 15)
Swallowing difficulty (%)	23% (*n* = 36)	29% (*n* = 8)
Continence problems (%)		
Bowel control	16% (*n* = 25)	29% (*n* = 8)
Bladder control	18% (*n* = 28)	36% (*n* = 10)
Cognition difficulty (%)		
Planning	56% (*n* = 88)	64% (*n* = 18)
Short term memory	70% (*n* = 111)	75% (*n* = 21)
Concentration	82% (*n* = 129)	82% (*n* = 23%)
Psychological problems (%)		
Depression	70%	75%
Anxiety	92%	90%

Abbreviations: COVID‐19, coronavirus disease 2019; ICU, intensive care unit; PCS, post‐COVID‐19 syndrome; *SD*, standard deviation.

Overall, 10% (*n* = 19) of the sample required telephone support from the AHP researcher or clinical team to complete the C19‐YRS due to a variety of factors (lack of digital access/digital literacy, cognitive impairment or fatigue levels, or a wish to quicken registration time by completing immediately). The length of time to administer the C19‐YRS by telephone with researchers was 30 min. It is unknown how long it took patients to complete independently at home; however, subsequent Patient and Public Involvement work demonstrated that it could be completed independently also within 30 min.

Patients' scores on the C19‐YRS sub‐scales are presented in Table 2. Fatigue was the most common complaint, with 97.3% of patients reporting fatigue of varying severity, followed by the onset of pain, which was not present before COVID‐19 was contracted (94.3%). The most common new pain was muscle pain, which affected 70% of patients, followed by headache (67%), chest (64%), and joint pain (59%). Approximately one‐third of patients also experienced new pain in their abdominal or other regions. Mental health problems were reported by 41% of patients, with 17% of these patients reporting respiratory or cardiac comorbidity. Respiratory or cardiac health issues, or both, were reported by 37% of patients. Swallowing, incontinence, skin rash, and fever were troublesome for very few the respondents.

**Table 2:** Patients' scores on the C19‐YRS sub‐scales

Subscale (scale range)	Valid scores	Mean (*SD*)	Median (IQR)	Score range	Skewness
Symptom severity (0–100)	125	42.7 (0.36)	40.0 (31.0–54.5)	10–81	0.232
Functional disability (0–50)	153	18.8 (10.7)	17 (11.0–26.5)	0–48	0.535
Additional symptoms (0–60)	155	18.8 (10.8)	18.0 (10.0–28.0)	0–48	0.246
Overall health (0–10)	183	4.6 (2.1)	4.0 (3.0–6.0)	0–10	0.265

*Note*: Data are only presented for patients with complete subscale scores.

Abbreviations: C19‐YRS, COVID‐19 Yorkshire Rehabilitation Scale; COVID‐19, coronavirus disease 2019; IQR, interquartile range; *SD*, standard deviation.

### 3.2 Data quality

Missing data for items were low (range 0.5%–19.8%). Subscale scores could be calculated for 67% of patients reporting symptom severity, 82% of patients reporting functional disability, 83% of patients reporting additional symptoms, and 98% of patients reporting overall health. Details of scores are given in Table 2.

### 3.3 Scaling assumptions

Item response option frequency distributions were symmetric. Item means and standard deviations were similar indicating that they were roughly parallel (Table 3), although there was a greater range in symptom severity. Corrected item‐total correlations exceeded 0.30 for all items except swallowing (0.24), incontinence (0.28), and skin rash (0.14), indicating that scaling assumptions were met for most items, including fever (0.33).

**Table 3:** Psychometric properties of the C19‐YRS sub‐scales

	Symptom severity	Functional disability	Additional symptoms	Overall health
Scaling assumptions				
Item means: range	0.9–7.2	3.5–4.9	3.5–4.6	4.0–4.9
Item *SD*: range	1.9–3.3	0.3–1.5	1.1–1.6	0.8–1.1
Item‐total correlations	0.24–0.62	0.39–0.67	0.16–0.62	
Targeting				
Missing data (%): range	0.5–19.8	0.5–15.5	5.9–12.3	2.1
Floor effects (%): range	5.3–72.7	16.4–61.0	15.0–66.8	2.1
Ceiling effects (%): range	0.0–9.6	0.5–4.8	0.0–10.2	1.1
Reliability				
Cronbach's *α*	0.79	0.79	0.70	

Abbreviations: C19‐YRS, COVID‐19 Yorkshire Rehabilitation Scale; COVID‐19, coronavirus disease 2019; *SD*, standard deviation.

### 3.4 Targeting

Scores spanned the range of the scale on admission and discharge and demonstrated good variability (Table 3). Results for some items demonstrated notable floor effects, especially for swallowing (72.7%), skin rash (66.8%), and fever (64.7%). There were no ceiling effects in any subscale.

### 3.5 Reliability

Internal consistency of the overall C19‐YRS was good (Cronbach's *α* = 0.891). Individual subscales also demonstrated good reliability. Deletion of the items noted to have poor scaling assumptions and targeting improved the reliability of the symptom severity subscale (swallowing, incontinence removed; Cronbach's *α* 0.79–0.81) and the additional symptoms subscale (fever, skin rash removed; Cronbach's *α* 0.70–0.74). These items were infrequently endorsed by participants, with fewer than 10% of participants endorsing a score greater than zero for any of these items, indicating that although bothersome to a small number of patients, their contribution to the overall measurement properties of the scale was limited.

The symptom severity, functional disability, and additional symptoms sub‐scales correlated strongly with each other (Table 4), indicating that the sub‐scales have a coherent internal structure. The overall health scale also correlated strongly with the other three subscales. As this is a more generic question of health status, this provides preliminary evidence of construct validity.

**Table 4:** Correlation of the C19‐YRS sub‐scales with the overall health scale*

	Pearson's correlation (significance) across subscales		
	Symptom severity	Functional disability	Additional symptoms
Overall health	−0.322 (<0.001)	−0.352 (<0.001)	−0.208 (0.010)
Additional symptoms	0.657 (<0.001)	0.515 (<0.001)	
Functional disability	0.772 (<0.001)		

Abbreviations: C19‐YRS, COVID‐19 Yorkshire Rehabilitation Scale; COVID‐19, coronavirus disease 2019.

*Overall health was reversed scored compared to item severity, so that an overall health score of “10” reflected the best possible health, in contrast to item severity where “10” reflected the worst possible severity of the symptom.

## 4 DISCUSSION

The C19‐YRS was developed by a rehabilitation research team as a disease‐specific patient‐based measure of the impact of COVID‐19 infection as part of the establishment of clinical service to meet the needs of patients recovering from the effects of the infection.^20^, ^21^ The scale has been used successfully to gather symptom severity and functional impact and monitor progress in PCS, and is recommended by the UK's National Health Service England,^22^ and the National Institute for Health and Care Excellence (NICE).^23^ However, it is recognized that the C19‐YRS requires further iterations for development and refinement, and wider demographic data to establish more of the determinants of the impact of COVID‐19 infection on individuals.

Many studies of rehabilitation in PCS have used generic measures of health outcomes. Conceptually, however, there are good arguments for making a PCS‐specific scale given that many rehabilitation strategies aim to ameliorate the specific impairments associated with PCS. We examined this self‐report version of the C19‐YRS, initially designed for use with patients discharged from acute hospital settings, then modified to suit both hospitalized and nonhospitalized patients, to determine the stability of the psychometric properties and its potential as a measure of PCS. In this first round of preliminary testing, our results provide evidence for that potential. In the group studied, evidence was found for data quality, scaling assumptions, targeting, and reliability. The findings from this study provide useful information and illustrate the potential of the C19‐YRS to achieve the necessary standards for highly accurate, psychometrically robust measurement.

This study has limitations. First, it is a study from a single clinical site and includes patients with a diverse range of experiences of acute COVID‐19 infection. While there is some evidence that small samples provide useful reliability and validity estimates,^24^ we recognize that our sample is relatively small at present. Nevertheless, our patient cohort is growing rapidly, and we aim to have in excess of 500 patients in our definitive psychometric analyses. Second, the scale is self‐report and thus the extent to which it is applicable in patients with severe fatigue or who have impairments affecting communication remains to be determined. In this study, patients could be provided with assistance to complete the questionnaire but is recognized that patients may answer items in questionnaires differently when the measures are administered by self‐completed questionnaire compared to an interview by a member of staff, and this may lead to a bias in the reporting of the scores.^25^ Third, we have not studied test‐retest reliability. However, Cronbach's alpha is considered to be a conservative reliability estimate, and test‐retest reliability often over‐estimates reliability. The underpinning research has been discussed by Nunnally^26^ and others.^15^, ^25^, ^26^

Despite these limitations, we are confident that the C19‐YRS will turn out to be a useful addition to current assessments of post‐COVID‐19 in clinical studies, and could be used to complement clinician‐rated measures of symptoms. Furthermore, the items in the scale provide qualitative information to clinicians to assist in targeting their clinical interventions to individuals' needs. It has advantages over other approaches, as it may be used in any setting, does not require an external rater, and is not laboratory‐based or require special equipment. Most importantly it measures patients' perspectives.^27^

### 4.1 Further research

In future validations, as cases accumulate, the researcher will seek outpatients whose circumstances and perspectives provide a contrast to those already included to achieve maximum variety in clinical, social, ethnic, and personal circumstances and health/digital literacy.

Subsequent psychometric testing will use Rasch analysis to determine whether the scale meets the fundamental axioms that define scientific measurement and permit the transformation of raw (ordinal) scores to interval level measurement.^11^ Further evaluations will examine the short‐ and long‐term responsiveness of the scale to changes in symptom severity and the overall impact of rehabilitation on PCS. This will also determine the minimal clinical important difference of the scale that correlates to clinical improvement or deterioration of the condition reported by patients.

## 5 CONCLUSION

This is the first study to examine the psychometric properties of a PCS‐specific outcome measure that captures and evaluates the symptoms experienced by patients. In this sample of patients, the C19‐YRS was clinically useful and satisfied standard psychometric criteria. The C19‐YRS shows good internal consistency, and scaling and targeting assumptions were satisfied. This provides preliminary evidence that the C19‐YRS outcome measure of PCS patients has satisfactory psychometric properties.

## CONFLICT OF INTERESTS

The authors declare that there are no conflict of interests.

## AUTHOR CONTRIBUTIONS

**Rory J. O'Connor** was the lead author responsible for the majority of the psychometric analyses and writing of the manuscript. **Nick Preston** conducted the initial statistical analyses, wrote the first and final draft of the paper, and addressed the reviewer's comments. **Amy Parkin, Sophie Makower, Denise Ross**, and **Manoj Sivan** were responsible for the data collection and organization, and contributed to writing the final drafts of the manuscript. **Mike Horton** is the senior psychometrician in the team and oversaw the overall psychometrics, provided field codes for data collection, and contributed to the writing of final drafts. **Stephen J. Halpin, Jeremy Gee**, and **Manoj Sivan** were responsible for the concept of the C19‐ Yorkshire Rehabilitation Scale, its original development, and contributed to the writing of final drafts.

## Data Availability

Data are available from the corresponding author upon reasonable request.
